# Overexpression of *NPR1* in *Brassica juncea* Confers Broad Spectrum Resistance to Fungal Pathogens

**DOI:** 10.3389/fpls.2017.01693

**Published:** 2017-10-04

**Authors:** Sajad Ali, Zahoor A. Mir, Anshika Tyagi, Hailay Mehari, Rajendra P. Meena, Javaid A. Bhat, Prashant Yadav, Pradeep Papalou, Sandhya Rawat, Anita Grover

**Affiliations:** ^1^National Research Centre on Plant Biotechnology, New Delhi, India; ^2^Centre of Research for Development, University of Kashmir, Srinagar, India; ^3^Division of Genetics, Indian Agricultural Research Institute, New Delhi, India; ^4^School of Bioengineering, SRM University, Chennai, India

**Keywords:** *Brassica juncea*, *NPR1*, *Alternaria*, *Powdery mildew*, systemic acquired resistance, salicylic acid, jasmonic acid

## Abstract

*Brassica juncea* (Indian mustard) is a commercially important oil seed crop, which is highly affected by many biotic stresses. Among them, *Alternaria* leaf blight and powdery mildew are the most devastating diseases leading to huge yield losses in *B. juncea* around the world. In this regard, genetic engineering is a promising tool that may possibly allow us to enhance the *B. juncea* disease resistance against these pathogens. *NPR1* (non-expressor of pathogen-related gene 1) is a bonafide receptor of salicylic acid (SA) which modulates multiple immune responses in plants especially activation of induced and systemic acquired resistance (SAR). Here, we report the isolation and characterization of new *NPR1* homolog (*BjNPR1*) from *B. juncea.* The phylogenetic tree constructed based on the deduced sequence of *Bj*NPR1 with homologs from other species revealed that *BjNPR1* grouped together with other known *NPR1* proteins of Cruciferae family, and was nearest to *B. napus*. Furthermore, expression analysis showed that *BjNPR1* was upregulated after SA treatment and fungal infection but not by jasmonic acid or abscisic acid. To understand the defensive role of this gene, we generated *B. juncea* transgenic lines overexpressing *BjNPR1*, and further confirmed by PCR and Southern blotting. The transgenic lines showed no phenotypic abnormalities, and constitutive expression of *BjNPR1* activates defense signaling pathways by priming the expression of antifungal *PR* genes. Moreover, *BjNPR1* transgenic lines showed enhanced resistance to *Alternaria brassicae* and *Erysiphe cruciferarum* as there was delay in symptoms and reduced disease severity than non-transgenic plants. In addition, the rate of disease spreading to uninfected or distal parts was also delayed in transgenic plants thus suggesting the activation of SAR. Altogether, the present study suggests that *BjNPR1* is involved in broad spectrum of disease resistance against fungal pathogens.

## Introduction

Plants are very often exposed to a variety of biotic stresses, and thus have evolved multidimensional defense approaches to survive or retain their fitness (Roux et al., 2014). The plants display both preformed and inducible defense mechanisms to overcome pathogen challenges. However, much stronger and long lasting is inducible defense response such as systemic acquired resistance (SAR), which confers enhanced disease resistance to broad range of phytopathogens (Durrant and Dong, 2004). In plants, SAR is generally activated by local infections and immunizes the whole plant to subsequent infectious diseases (Fu and Dong, 2013; Shah and Zeier, 2013). Realistic evidences have shown that activation of SAR is reliant on the higher levels of the endogenous salicylic acid (SA), and activation of a battery of pathogen-related (*PR*) genes. Most of these PR proteins such as glucanases, chitinases, thaumatins, and defensins possess antifungal activities and are known to play important role in disease resistance. Exogenous application of SA or its analogs have been also revealed to activate SAR pathway in plants (Durrant and Dong, 2004; Makandar et al., 2006). Conversely, *Arabidopsis thaliana* plants expressing *NahG* transgene which codes for salicylate hydroxylase (SA-degrading enzyme) were deficient in accumulating SA, and hence failed to activate SAR (Delaney et al., 1995). In addition to SA, a group of heterogeneous proteins are crucial for the activation of SAR. Among them are the NPR1 protein, a key regulator in the SA mediated SAR signal transduction pathway. The quest to discover the SA receptor led to the discovery of a regulatory or transcription co-factor protein NPR1 (Cao et al., 1994). However, many studies have revealed that *NPR1* is linked to SA signaling, however, its role as SA receptor remains largely unknown. In this context, Wu et al. (2012) has recently reported that NPR1 is the receptor for SA pathway in *Arabidopsis*. In addition, two *NPR1* paralogs namely, *NPR3* and *NPR4* bind SA and control the proteasome-mediated degradation of NPR1 protein through their interaction with *NPR1* (Fu et al., 2012).

After pathogen assault, plants produce a variety of phytohormones, their composition, quantity, and timing significantly varies among plant species, and depends mainly on the pathogens lifestyle and their mode of infection (De-Vos et al., 2005). SA pathway generally provides resistance to biotrophic pathogens, whereas jasmonic acid/ethylene (JA/ET) pathways are commonly associated with resistance to necrotrophic pathogens, and to herbivorous pests (Glazebrook, 2005; Bari and Jones, 2009). Generally, SA and JA signaling pathways operates antagonistically and thus, elevated resistance against biotrophs is often related with increased susceptibility to necrotrophs, and *vice versa* (Grant and Lamb, 2006). Many regulatory components involved in SA/JA crosstalk have been identified; among them is NPR1 which plays a crucial role in regulating SA-mediated suppression of the JA pathway (Spoel et al., 2003; Pieterse et al., 2012; Thaler et al., 2012; Van der Does et al., 2013). Furthermore, SA/JA antagonism is commonly found in many plant species under various taxonomic groups, therefore seems to be evolutionary conserved evolutionary (Thaler et al., 2012).

As first discovered in *Arabidopsis*, various *AtNPR1* homologs have been isolated thereafter in many agriculturally important crops (Chen et al., 2013; Zhong et al., 2015). *NPR1* is a multigene family in *Arabidopsis* with multifaceted functions. For example, *AtNPR1* and *AtNPR2* are notably considered as a key regulator of SAR (Cao et al., 1997, 1998; Zhang et al., 2003) while *AtNPR3* and *AtNPR4* are known as negative regulator of SAR (Fu et al., 2012). Moreover, another group of *AtNPR1* homologs are *AtBOP1*, and *AtBOP2*, which are related with lateral organ development (Hepworth et al., 2005). However, most of the studies were carried out on *Arabidopsis NPR1* (*AtNPR1*). Structurally, *AtNPR1* and its homologs contain an ankyrin repeat, N-terminal BTB/POZ broad-complex, Tramtrack, Bric a brac/poxvirus and zinc finger domains, respectively (Cao et al., 1997; Aravind and Koonin, 1999). In non-induced state, *NPR1* exists as an inactive oligomer form into cytosolic region. However, after SA accumulation, the redox status of the cell changes which leads to dissociation of the inactive oligomer *NPR1* to active monomers and their translocation to the nucleus where they bind to TGA factors there by inducing *PR* genes (Mou et al., 2003; Tada et al., 2008). Transcriptional studies have shown that *NPR1* is expressed at low levels in mock plants but is induced significantly after microbial attack or treatment with SA or its biologically active analogs. Many studies have revealed *NPR1* mutant (*NPR1*) plants are more prone to diseases, and also show altered expression of defense marker *PR* genes compared to NPR1 expressing plants (Glazebrook et al., 1996; Cao et al., 1997). Furthermore, *NPR1* also plays a role in cross talk of SA/JA signaling pathways and in antagonistic effect of SA on JA signaling (Spoel et al., 2003). Hence, *NPR1* is considered as the positive regulator of SA mediated plant immune responses.

To explore the defense role of *NPR1* against bacterial, viral and fungal pathogens, various overexpression studies have been carried out in both model and crop plant systems. *NPR1* mediates the SA-induced expression of pathogenesis-related (PR) genes and SAR. Overexpression of *NPR1* results in the increase of transcript levels of antifungal genes like *PR1, PR2* (glucanase) and *PR5* (thaumatin) which are universally known to have antifungal activity. Many studies have revealed the potential antifungal activity of these *PR* genes against wide range of fungal pathogens. *PR* gene activity is regulated at the level of redox-dependent nuclear transport of NPR1. For example, overexpression of *NPR1* in *Arabidopsis* plants confers enhanced disease resistance to bacterial and fungal infections (Cao et al., 1998; Friedrich et al., 2001). Transgenic carrot plants overexpressing *AtNPR1* exhibit high disease resistance not only to biotrophs (*Erysiphe heraclei*) but also to necrotrophic fungal pathogen (*Botrytis cinerea, Sclerotinia sclerotiorum*, and *Alternaria radicina*), respectively (Wally et al., 2009). Parkhi et al. (2010) also reported that cotton transgenic plants expressing *AtNPR1* exhibited broad spectrum of disease resistance not only to fungal pathogens but also to nematodes. Additionally, tobacco plants overexpressing *Malus hupehensis NPR1* confers resistance to *Botrytis cinerea* as well as induces battery of pathogen related genes. Furthermore, studies revealed have that rice and wheat plants overexpressing *NPR1* gene confers broad spectrum of disease resistance against most disastrous pathogens *Magnaporthe oryzae, Fusarium verticillioides*, and *Fusarium oxysporum*, respectively (Makandar et al., 2006; Quilis et al., 2008). *NPR1* overexpression in *A. thaliana* was reported to result an increase in the transcript levels of *PR* genes, hence proves that *NPR1*-dependent *PR* gene mediated disease resistance (Cao et al., 1997; Friedrich et al., 2001). Most recently, overexpression of *NPR1* was also revealed to confer disease resistance against broad range of pathogens in different crops (Dutt et al., 2015; Sundaresha et al., 2016). These results revealed that *NPR1* is a potential candidate gene for developing disease resistant transgenic crops against multiple pathogens.

*Brassica juncea* is an economically important oilseed crop in India and fulfills nearly 27% of vegetable oil requirements of the country (Giri et al., 2013), but fungal diseases have emerged as devastating factors for its poor yield and economic losses (Bal and Kumar, 2014; Bairwa et al., 2015; Chandrashekar et al., 2015). In field conditions, *B. juncea* are challenged by many potential fungal pathogens such as *Alternaria brassicae* (*Alternaria* leaf blight), *Albugo candida* (white rust), *Sclerotinia sclerotiorum* (Sclerotinia stem rot), *Erysiphe cruciferarum* (powdery mildew), and *Hyaloperonospora parasitica* causative agent of downy mildew. Generally, *B. juncea* lack sufficient innate resistance to these fungal diseases and development of resistant varieties through conventional breeding is difficult due to unavailability of disease resistant germplasm. However, fungicides are one of the tools to control fungal diseases but are environmentally detrimental, expensive and ineffective due to weather fluctuations. In this regard, *B. juncea* being an important source of edible oil, novel strategies of effective disease protection should be developed. Therefore, genetic transformation of defense regulatory genes (like *NPR1*) that controls the function of multiple defense genes are potential sources for developing broad spectrum and long lasting disease resistance against pathogens in *B. juncea*. In this study, we evaluated the role of *NPR1* in *B. juncea* for improving disease resistance against biotrophic and necrotrophic fungal pathogens.

## Materials and Methods

### Plant Materials

The plant material used in the present study is *B. juncea* var. Varuna, and plants were grown in pots containing a mixture of soil and organic manure (2:1) in a growth chamber under a 16 h day/8 h dark photoperiod at a temperature of 22–24°C, with irradiance of 100–125 μmol/m^2^s and a relative humidity of 80%. For cDNA library construction, *B. juncea* plants were sprayed with 2 mM SA (pH 7.0) and control plants were similarly treated with sterile distilled water. Leaf samples for RNA isolation were harvested from control and SA treated plants at different time points.

### Isolation and *In Silico* Analysis of *NPR1* Gene from *B. juncea*

*Brassica juncea* cDNA library was constructed using total RNA (2 μg) from SA treated leaves as described (Ali et al., 2017). The full length cDNA of *BjNPR1* was isolated from *B. juncea* cDNA library through colony hybridization using radiolabeled *Arabidopsis NPR1* (*AtNPR1*) probe. Primers used to amplify *AtNPR1* probe are presented in **Table 1**. A BLAST homology search against the NCBI database was carried out to confirm whether the obtained sequence encoded *BjNPR1*. The multiple sequence alignment was performed using ClustalW^1^. To determine the evolutionary relationship of *BjNPR1* protein with other *NPR1* homologs from monocots and dicots, phylogenetic tree was constructed using MEGA 7.1 with 1000 bootstraps. Conserved domain structure of this protein was analyzed by Pfam database^2^. The 3D (three-dimensional) structure of *BjNPR1* was obtained using Phyre2 server^3^. Molecular weight and isoelectric point of *BjNPR1* protein were obtained using Compute PI/MW tool of ExPASy. *In silico* subcellular localization of this protein was predicted using Cell-PLoc 2.0 program^4^.

**Table 1:** List of primers used in this study.

Gene	Primer
*35SPro*	F-5′ CGGATTCCATTGCCCAGCTA 3′
*NPR1pro*	R-5′ GAGAGTGCTGCTTTGGTTGC 3′
*RT-NPR1*	F-5′ GGAAGGAGCCGAGTTTGATAG 3′ R-5′ GTTATACTCACCCGCCTTAGTG 3′
*NPTII*	F-5′ AGGCGATAGAAGGCGATGCGC 3′ R-5′ CAATCGGCTGCTCTGATGCCG 3′
*RT-PR1*	F-5′ GAACACGTGCAATGGAGAATG 3′ R-5′ CCATTGTTACACCTCGCTTTG 3′
*RT-PR2*	F-5′ CGTCTCTCTACAATTCGCTCTG 3′ R-5′ CGATATTGGCGTCGAATAGGT 3′
*RT-PR3*	F-5′ AAGACCAGGTTCTTGCCTTC 3′ R-5′ TCCGGTACACTCCCTACTATTC 3′
*RT-PR5*	F-5′ GCAGAACAATTGCCCTTACAC 3′ R-5′ GCGCCTGGATTCAGTTGATA 3′
*RT-PR12*	F-5′ CAATGGTGAAAGCGCAGAAG 3′ R-5′ AGGTTGATGCACTGGTTCTT 3′
*RTPR13*	F-5′ GAGAAGCAATGGCAGGTTCTA 3′ R-5′ CGCACTCCGTGTTGTAGTT 3′
Alpha tubulin	F-5′ GCCTCGTCTCTCAGGTTATTTC 3′ R-5′ TGAAGTGGATTCTTGGGTATGG 3′

### Expression Pattern of *BjNPR1* under Hormonal Treatments and Fungal Infection

To investigate the induction of *BjNPR1* gene, 40 days old *B. juncea* plants were sprayed with 2 mM SA, 100 μM JA and 50 μM abscisic acid (ABA) individually. Control plants for each treatment were treated with sterile distilled water containing equal amount of solvent used for hormone preparation. Leaf samples for RNA isolation were harvested from control and hormone treated plants after 0, 2, 4, 8, 12, 24, 48, and 72 h. For fungal infection, *A. brassicae* strain (I.D. No. 81651) was grown on radish dextrose agar (RDA) medium (Thakur and Kolte, 1985) at 22°C for 15 days and spores were collected to prepare inoculum. The 40 days old *B. juncea* plants were infected with *A. brassicae* as described by Ali et al. (2017). The inoculated plants were maintained at 100% relative humidity at 25°C. For powdery mildew infection, *E. cruciferarum* (H.C.I.O-ID: No.52067) previously isolated and identified in our lab was used as inoculum. Forty days old *B. juncea* plants were infected by physically dislodging conidia of *E. cruciferarum* from infected leaves on healthy leaves of *B. juncea* plants. The inoculated plants were kept in growth chamber at 100% relative humidity and 25°C. Control *B. juncea* plants were mock inoculated with sterile distilled water and incubated separately to prevent cross contamination. Leaf samples from control and fungal infected plants were harvested at different time points flash frozen in liquid nitrogen and stored at -80°C.

### RNA Isolation and Reverse Transcription Quantitative PCR

To analyze the expression of *BjNPR1* after hormonal treatments and fungal infection, reverse transcription quantitative PCR (RT-qPCR) was performed using *BjNPR1* gene specific primers. Total RNA was isolated from 100 mg of leaf sample collected from treated and control plants using Ambion RNA isolation kit as described by manufacturer’s protocol (Life Technologies). Complementary DNA (cDNA) was synthesized from 2 μg of purified total RNA by reverse transcriptase in 20 μl reaction volume containing oligo(dT) 18 primers, 10 mM deoxynucleotide (dNTPS) and water following the manufacturer’s instructions (Invitrogen, Canada). RT-qPCR reaction mixture contains 2 μl of cDNA, 5 μl of SYBR green real-time PCR master mix (Takara, Japan) and 0.5 μl (10 pmol) of each primer (*BjNPR1*). The RT-qPCR thermocycling program was following: 95°C for 5 min, followed by 40 cycles at 94°C for 30 s, at 60°C for 30 s, and at 72°C for 30 s. All primers used in this study were designed by Oligoanalyzer software (**Table 1**). The alpha *tubulin* gene (GenBank accession no-NM_100360.) was used as reference gene for normalization of expression values. The relative expression levels of *BjNPR1* were quantified by 2^-ΔΔCt^ method (Livak and Schmittgen, 2001). All reactions were conducted with three biological replicates. Fold changes with *p*-values less than 0.05 were considered significant.

### Binary Vector Construction and *Agrobacterium* Transformation

The full length cDNA of *BjNPR1* was cloned in sense direction into *pBI121* at Sma1 and Sac1 site downstream of constitutive promoter *35S* CaMV (cauliflower mosaic virus). The correct orientation of *BjNPR1* fragment in the recombinant plasmid was further identified by PCR and sequencing. The resulting recombinant binary vector *pBI121*-*BjNPR1* was mobilized into *Agrobacterium tumefaciens* EHA105 by freeze-thawing method (Holsters et al., 1978).

### *Brassica juncea* Transformation

*Brassica juncea* cv. Varuna seeds were germinated on half strength Murashige and Skoog (MS) medium in Magenta boxes (Magenta vessel Corp., United States) at 24 ± 2°C under cool white florescent light (90–150 μmol photons/m^2^s) in a 16/8 h (light/dark) photoperiod (Murashige and Skoog, 1962). *BjNPR1* transgenic plants were generated through *Agrobacterium* mediated co-cultivation method according to protocol with some modifications (Sharma et al., 2009). Seeds obtained from T_0_ transformed *B. juncea* plants were primary screened on kanamycin selection medium and then transferred into pots for further analysis.

### Molecular Screening of *BjNPR1* Transformants

Genomic DNA was isolated from putative *BjNPR1* transformed and non-transformed plants following CTAB method. For molecular screening of *BjNPR1* transgenic plants PCR and Southern blot analysis was used. PCR detection of *BjNPR1* transgene was carried out using *35S* promoter (forward) and *BjNPR1* (reverse) primers. Southern blot analysis was performed to detect the transgene insertion and the copy number using DIG High Prime DNA Labeling and Detection Starter Kit I (Roche Applied Science, Mannheim, Germany). For integration, a 600 bp fragment of *35S* promoter (forward primer) and NPR1 (reverse primer) were used as a probe in *BjNPR1* transgenics. To detect copy number of *BjNPR1* in transgenic plants, NPTII probe (500 bp) was used. Details of gene specific primers of *BjNPR1, 35S* promoter, and NPTII used for PCR analysis and probe synthesis are given in **Table 1**.

### Expression Analysis of *NPR1* and *PR* Genes in *BjNPR1* Transgenics

RT-qPCR was performed to monitor the transcript levels of the *BjNPR1, PR1, PR2* (β 1-3 glucanse), *PR3* (chitinase), *PR5* (thaumatin), *PR12* (defensin), and *PR13* (thionin) in the leaf tissue of the transgenic and non-transgenic plants. RNA isolation, cDNA synthesis and RT-qPCR experiments were executed as described in the above section. All primers used in this study are listed in **Table 1**.

### Phenotypic Characterization of *BjNPR1* Transgenic Plants

Different agronomic traits namely, size and shape of leaf, siliques, flowers, number of pods, number of seeds as well plant height were investigated for any phenotypic abnormalities between *BjNPR1* transgenic and wild-type *B. juncea* plants.

### Necrotrophic and Biotrophic Resistance Assay

*BjNPR1* transgenic lines were evaluated for disease resistance to both necrotrophic and biotrophic fungal pathogens. *Alternaria* infection in *BjNPR1* transgenic lines and non-transformed plants were carried out as described in our previous work (Ali et al., 2017). For disease scoring, three components of partial resistance, including lesion appearance, number of lesions per leaf, lesion diameter (mm) and percentage of disease leaf area (%DLA) were measured and compared between *BjNPR1* transgenic lines and control plants after inoculation. Disease severity was calculated after 15 days inoculation (DAI), with a 10-point disease rating scale. For, *E. cruciferarum* infection, 40 days old healthy *BjNPR1* transgenic and wild-type *B. juncea* plants were infected as described in above section. The inoculated plants were maintained at 22°C with 100% RH in an inoculation chamber. Disease phenotype was examined at 7 days after powdery mildew inoculation. For disease scoring, different parameters were used such as colony appearance, number of colonies or spots, disease index, percentage disease leaf area (%DLA) between *BjNPR1* transgenic lines and control plants. Disease index including six grades: 0, 1, 3, 5, 7, and 9, which correspond, respectively, to disease incidence levels of 0, ≤5%, 6–10%, 11–20%, 21–40%, and ≥41%). The experiments were carried out in three biological replicates.

### Trypan Blue Staining and Microscopy

Trypan blue staining was used for observing dead cells and fungal biomass in control and *BjNPR1* transgenic plants. Briefly, control and transgenic infected leaves were stained with trypan blue staining solution [containing 40 mg of trypan blue, 10 mL lactic acid (85% w:w), 10 mL phenol (pH 7.5–8.0), 10 mL glycerol (≥99%) and 10 mL of distilled water] for 30 min at room temperature. The samples were washed with sterile water to remove the excess stain and then immersed in 70% ethanol solution overnight to remove the chlorophyll. The solution was then removed and tissue samples were immersed in 60% glycerol and photographed. The fungal biomass and cell death was visualized under light microscope (10 xs, Nikon).

### Statistical Analysis

For all experiments, three biological replicates were used and each repeated three times. A student’s *t*-test was carried out to determine significant differences in *BjNPR1* gene expression in control and treated samples as well transgenic and non-transgenic plants. The differences between two groups of data for comparisons in all the experiments were evaluated as statistically significant (^∗^*p* < 0.05) or extremely significant (^∗∗^*p* < 0.01).

## Results

### Isolation and *In Silico* Analysis of *BjNPR1*

**FIGURE 1:**
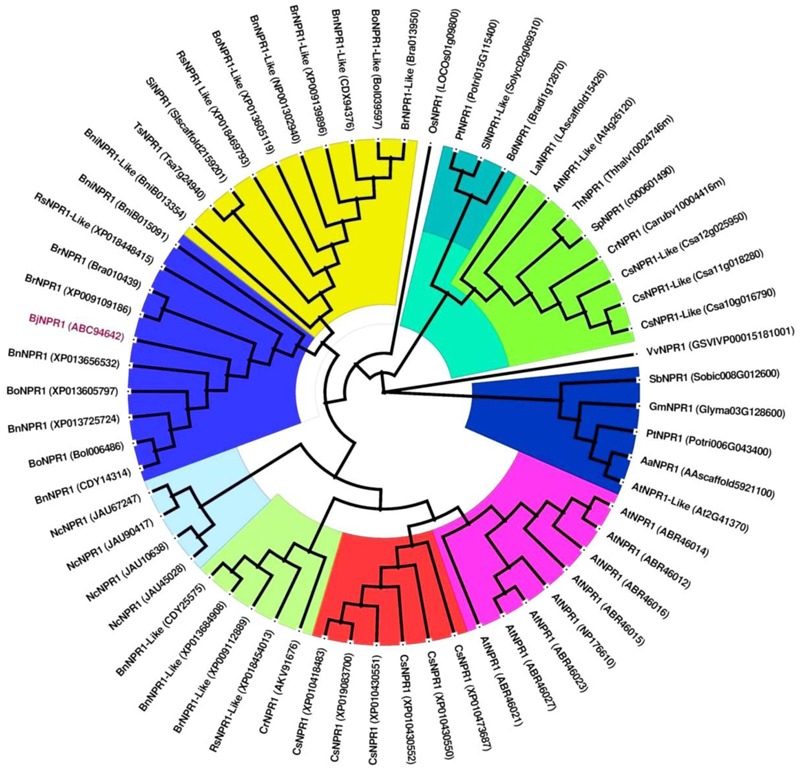
Phylogenetic analysis of BjNPR1 with other NPR1 proteins from different plant species. The deduced amino acid sequences of BjNPR1 was retrieved from NCBI GenBank and were further aligned with ClustalW using MEGA7.1 bioinformatic tool. The tree was generated using Maximum-Likelihood (ML) method with 1000 bootstrap replicates. GenBank IDs of each NPR1 protein sequence are given in the brackets behind the species names.

**FIGURE 2:**
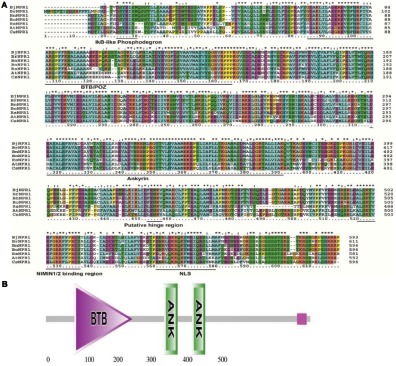
A multiple sequence alignment and *in silico* analysis of BjNPR1 protein sequence. **(A)** Alignments of the deduced amino acid sequences of BjNPR1 (accession no. ABC94642) revealed 92% identity with BnNPR1 (accession no. XP013725724), 78% with BolNPR1 (accession no. XP013605797), 78% with BrNPR1 (accession no. XP009109186), and 66% with AtNPR1 (accession no. ABR46023). **(B)** The conserved domains (BTB-ANK) of the BjNPR1 protein after analyzed by Pfam.

The full-length cDNA of *BjNPR1* gene was isolated from SA treated *B. juncea* library, and submitted to the Genbank with accession number DQ359129. *In silico* analysis of *BjNPR1* cDNA revealed that it is comprised of 1781 bp with an open reading frame of 1857 bp, encoding a protein of 593 amino acids with a molecular mass of 65.77 kDa, and a theoretical PI of 5.25. Phylogenetic analysis showed that *BjNPR1* is very close homolog to NPR1 of *B. napus, B. oleracea, B. rapa*, and *A. thaliana*, respectively, but was largely diverged from NPR1 of Poaceae family (**Figure 1**). Alignment of deduced amino acid sequence of BjNPR1 (accession no. ABC94642) revealed 92% identity with BnNPR1 (accession no. XP013725724), 78% with BolNPR1 (accession no. XP013605797), 78% with BrNPR1 (accession no. XP009109186), and 66% with *AtNPR1* (accession no. ABR46023) (**Figure 2A**). To investigate the typical domain structure of BjNPR1, its protein sequence were analyzed using Pfam software which revealed the predicted BTB, ANK conserved domain as shown in **Figure 2B**.

### Expression Analysis of *BjNPR1* in *B. juncea* after Hormonal Treatments and Fungal Infection

**FIGURE 3:**
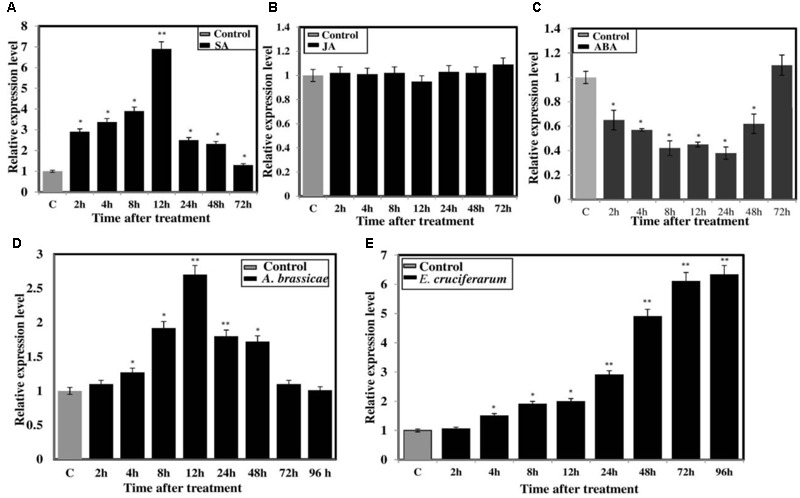
Expression analysis of *BjNPR1* gene after hormonal treatment and fungal infection. Forty days old *B. juncea* plants were treated with different defense stimulators (SA, JA, and ABA) and also infected with fungal pathogens (*A. brassicae and E. cruciferarum*). Leaf samples were harvested at different time points for RNA isolation. Control plants for each treatment were treated with sterile distilled water containing equal amount of solvent used for hormone preparation. **(A)** Expression analysis of *BjNPR1* after SA **(B)** JA **(C)** ABA **(D)** *Alternaria* infection and **(E)** *E. cruciferarum*, respectively. SE for each bar is shown. The asterisks indicate statistically significant difference relative to control and was calculated by student’s *t*-test (^∗^*P* < 0.05; ^∗∗^*P* < 0.01).

The expression pattern of *BjNPR1* was investigated under various hormonal stresses (SA, JA, and ABA) as well as inoculation with *A. brassicae* and *E. cruciferarum* through real-time PCR. Upon SA treatment, transcript levels of *BjNPR1* started to increase at 2 h (2.9-fold) and reached a peak at 12 h with a sharp decline at later time points (**Figure 3A**). On the other hand, no significant induction of *BjNPR1* was seen in JA-treated plants and remains the same as control (**Figure 3B**). Treatment of *B. juncea* plants with ABA decreases the transcript levels of *BjNPR1* at 2 h (0.65-fold) and remained low until 72 h time interval (**Figure 3C**). It has been well documented that *NPR1* plays critical role in disease resistance in plants. To further study the defensive role of *BjNPR1*, we inoculated *B. juncea* plants with both necrotrophic (*A. brassicae)* and biotrophic (*E. cruciferarum*) fungal pathogens. After *Alternaria* inoculation, the expression of *BjNPR1* was slightly increased at 4 h, reaching maximum at 12 h (2.7-fold) of post inoculation (**Figure 3D**). On the other hand, inoculation of *B. juncea* plants with *E. cruciferarum* led higher up-regulation of *BjNPR1*, and the highest expression levels were observed after 72 h (6.11-fold) to 96 h (6.33-fold) compared to control (**Figure 3E**). Hence, these results suggest that *BjNPR1* is induced by fungal pathogens and might play important role in *B. juncea* disease resistance.

### Development and Molecular Analysis of Transgenic *B. juncea* Lines Overexpressing *BjNPR1*

**FIGURE 4:**
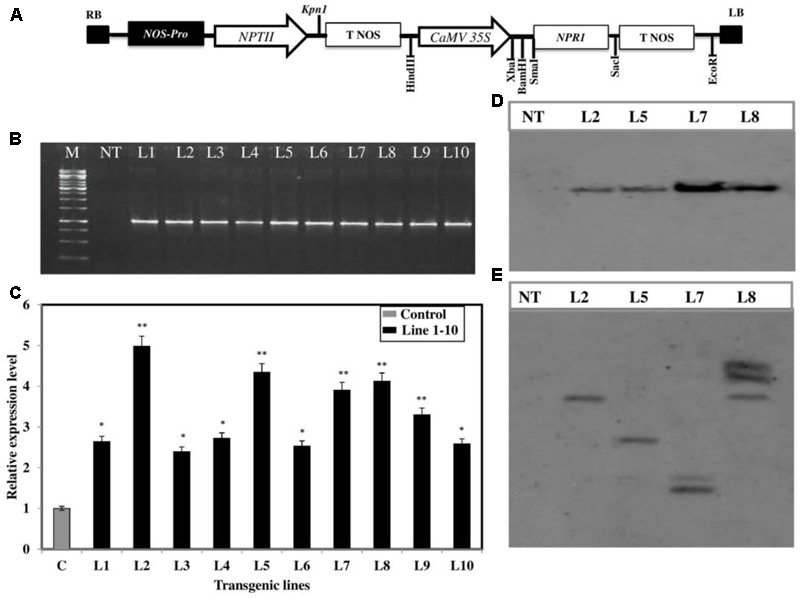
Development of *35S*-*BjNPR1* transgenic lines and molecular analysis. **(A)** Schematic representation of *BjNPR1*-pBi121 construct and T-DNA map of pBi121 binary vector. **(B)** T-DNA integration in 10 transgenic lines was confirmed by PCR amplification, using *35S* promoter (forward) and *BjNPR1* (reverse) gene specific primers. **(C)** Real-time PCR quantification of *BjNPR1* in wild type and *35S-NPR1* overexpressed *B. juncea* plants. The asterisks indicate statistically significant differences between control (non-transgenic) and *BjNPR1* transgenic plants (^∗^*P* < 0.05; ^∗∗^*P* < 0.01). **(D)** Southern blotting analysis showing *BjNPR1* transgene integration, DNA was digested with *Hind*III restriction enzyme and hybridized with DIG labeled *35S-NPR1* probe. **(E)** Southern blotting analysis showing copy number in selected highly expressed transgenic line (L2, L5, L7, and L8), respectively, DNA was digested with *Sac*1 and hybridized with DIG labeled NPTII gene probe.

In order to further confirm the defensive role of the *BjNPR1*, transgenic *B. juncea* lines with constitutive expression of *BjNPR1* were generated through tissue culture method to enhance the immunity (**Supplementary Figures S1A–J**). For this purpose, full length cds of *BjNPR1* was cloned into pBI121 binary construct under the control of CaMV *35S* constitutive promoter (**Figure 4A**). Further, *BjNPR1* transgenic plants were generated using *Agrobacterium* hypocotyls co-cultivation method (**Supplementary Figure S1**). In the present study, overall transformation efficacy was found to be 2.7% using co-cultivation method (Supplementary Table S1). A total of 10 transgenic lines were obtained through kanamycin screening, and T-DNA integrations were confirmed by PCR amplification (**Figure 4B**). Overexpression of *BjNPR1* was examined in transgenic lines by q-RT PCR. The mRNA levels of the *BjNPR1* gene varied greatly in different lines such as lines 2, 5, 7, and 8 showed high accumulation of *BjNPR1*, while remaining lines (1, 3, 4, 6, 9, and 10) revealed relatively low expression levels of *BjNPR1* (**Figure 4C**). To further investigate the integration and copy number of *BjPR1* transgene in selected highly expression lines (2, 5, 7, and 8), Southern blotting was performed using *35S-NPR1* and *NPTII* probes, and all the four lines showed *35S*-*BjNPR1* integration, respectively (**Figure 4D**). In addition, single copy insertion was observed in T-DNA lines 2 and 5, where as two copies were found in lines 7 and 8 (**Figure 4E**). Finally, single copy number and high expression lines of *BjNPR1* transgenic (lines 2 and 5) were selected for disease screening.

### *BjNPR1* Transgenic Plants Modulates the Expression of SA and JA Signature (*PR*) Genes

**FIGURE 5:**
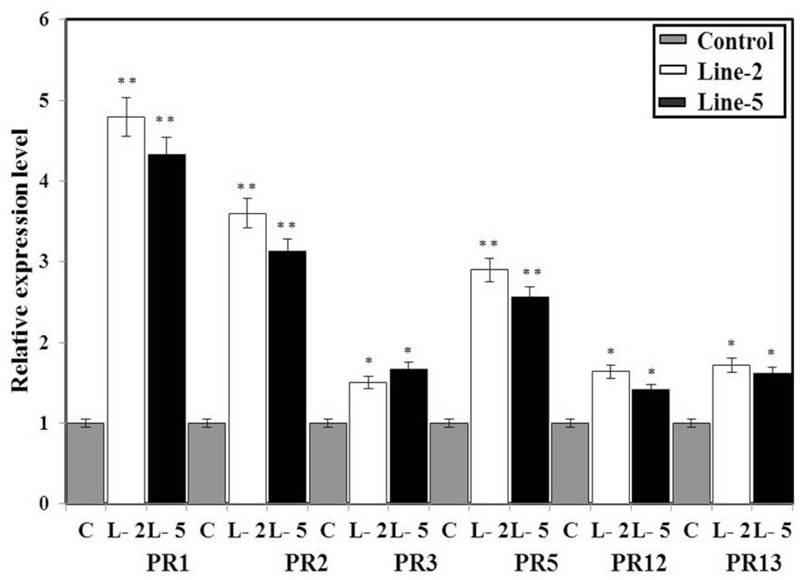
Quantification of mRNA levels of SA- and JA-dependent *PR* genes in wild type and *BjNPR1* transgenic plants. Expression analysis of *PR1, PR2, PR3, PR5, PR12*, and *PR13* in control and *BjNPR1* transgenic plants. The relative expression levels of SA and JA dependent *BjPR* genes in transgenic and wild-type plants were compared with that of a control alpha *tubulin* gene. The data are the mean ± SE of three biological replicates. SE for each bar is shown. The asterisks indicate statistically significant differences between the *BjNPR1* transgenic and control (non-transgenic) plants (^∗^*P* < 0.05; ^∗∗^*P* < 0.01).

Pathogen-related genes are not only known as molecular signatures of the SA and MeJA signaling pathways but also widely used as diagnostic markers in pathogen resistance assays. Generally, overexpression of *NPR1* genes has been shown to enhance the immune response (so called priming) through the activation of SAR marker or *PR* genes. Here, we examined whether *B. juncea* plants overexpressing *BjNPR1* gene will lead to induction of *PR* genes. For this, we studied the expression of SA and JA signaling diagnostic genes (*PR1, PR2, PR3, PR5, PR12*, and *PR13*) in selected highly expressed *BjNPR1* transgenic lines and non-transgenic plants under non-stressed conditions. The expression levels of SA marker genes *PR1, PR2*, and *PR5* in *BjNPR1* transgenic lines (L2 and L5) was found to be comparatively higher than in control (non-transgenic) plants (**Figure 5**). On the other hand, low expression levels of JA signature genes (*PR3, PR12*, and *PR13*) were observed in *BjNPR1* transgenic lines when compared to SA signature genes but were higher than in control (non-transgenic) plants. These results showed that the constitutive expression of *BjNPR1* was associated with the faster and stronger activation of *PR* genes which could enhance disease resistance in *B. juncea* to multiple pathogens.

### Phenotypic Analysis of *BjNPR1* Transgenic Plants

**FIGURE 6:**
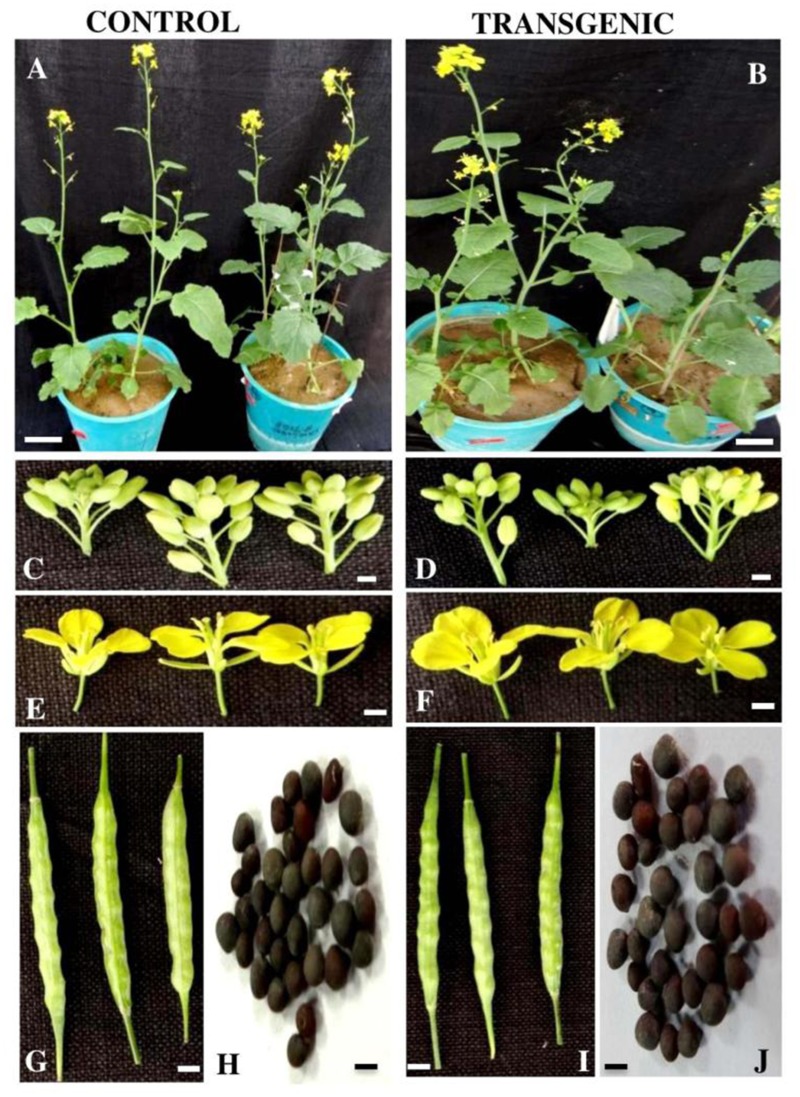
Agronomic traits of wild type and *BjNPR1* transgenic plants. Different agronomic traits such as size and shape of leaf, silique, flower, number of pods, number of seeds as well plant height were studied in *BjNPR1* transgenic and control plants. *BjNPR1* transgenic lines did not show any phenotypic abnormalities. Scale bar 1 cm **(A,B)**, 0.1 cm **(C,D)**, 0.5 cm **(E,F)**, 0.1 cm **(G,I)**, and 0.05 cm **(H,J)**.

In this study, comparative analyses on phenotypic abnormalities in *BjNPR1* transgenic plants were systematically evaluated. Our results revealed that all the studied agronomic traits namely, shape and size of leaves, siliques, flower morphology, seed shape, number of pods, number of seeds and plants height in *BjNPR1* transgenic plants were similar with that of wild-type plants (**Figure 6** and **Table 2**). Hence, these results provides the evidence that *BjNPR1* transgenic lines did not show any phenotypic abnormalities as was observed in other crop plants after overexpressing *AtNPR1*. Altogether, this data indicated that *BjNPR1* transgenic plants showed normal growth and development.

**Table 2:** Phenotypic analysis of *BjNPR1* transgenic lines and wild-type plants did not show significant variations in terms of plant height, no of seeds and pods.

S. No.	Plants	Plant height (cm)	No. of seeds/pod	No. of pods/plant
1	C	134.3 ± 1.5	13 ± 0.3	77 ± 0.3
2	L2	135.7 ± 1.5	12 ± 0.3	76 ± 1.8
3	L5	135 ± 1.2	12 ± 0.6	78 ± 0.7

### Overexpression of *BjNPR1* in *B. juncea* Transgenic Plants Confers Partial Disease Resistance to Necrotrophic Fungal Pathogen

**FIGURE 7:**
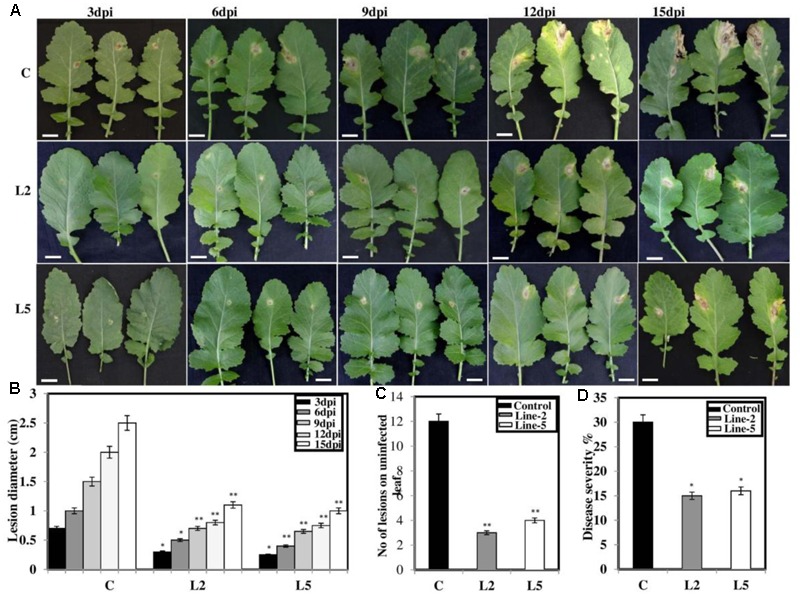
Overexpression of *BjNPR1* improves disease resistance in *B. juncea.* **(A)** Disease resistance screening of *BjNPR1* transgenic lines (L2 and L5) after *Alternaria* infection. L2 and L5 showed delay and reduction in disease severity than wild-type plants. **(B)** Lesions diameter of control (wild type), L2 and L5 *BjNPR1* lines after *Alternaria* infection at different dpi. Bar = 40 mm. **(C)** Evaluation of disease resistance to *Alternaria* in *BjNPR1* transgenic lines using six-point disease severity index. **(D)** *Alternaria* disease severity was monitored in *BjNPR1* transgenic lines and control plants at 15th dpi based on total leaf area infected. Three biological replicates were used for infection. SD are the means of three biological replicates and asterisks shows statistically significant difference (^∗^*P* < 0.05; ^∗∗^*P* < 0.01) between *BjNPR1* transgenic lines and wild-type plants.

**FIGURE 8:**
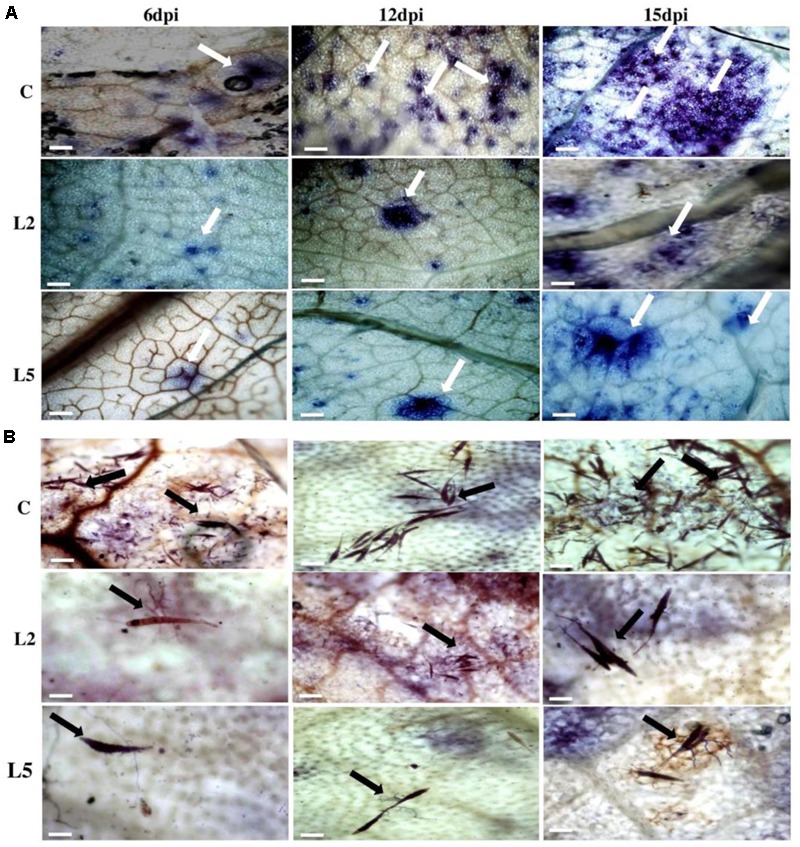
Analysis of cell death and fungal biomass in *BjNPR1* transgenic and wild-type plants using trypan blue staining. **(A)** Microscopic examination of *Alternaria* mediated cell death in *BjNPR1* lines and wild-type plants are shown with white color arrows. **(B)** Trypan blue staining of *BjNPR1* transgenic and control plants after *Alternaria* infection. *Alternaria* spore load or biomass in *BjNPR1* transgenic lines and wild-type plants at various dpi are shown with black color arrows. *BjNPR1* transgenic lines showed reduced cell death and spore count as compared to wild-type plants. Bar = 40 μm.

To explore the role of *BjNPR1* in disease resistance, we evaluated resistance level in *BjNPR1* transgenic plants against necrotrophic (*A. brassicae*) fungal pathogen, which is the most serious pathogen of *B. juncea*. We selected two transgenic lines (L2 and L5) for this study based on expression levels of *BjNPR1* transgene. For *Alternaria* infection, *BjNPR1* transgenic and control plants were infected and disease scoring was assessed at different time intervals. After inoculation, small necrotic lesions began after 3 days in non-transgenic plants and size of the necrotic lesions increased significantly after disease progression. In contrast, *BjNPR1* transgenic plants also showed lesions but the lesion size or diameter was comparatively lower than non-transgenics (**Figure 7A**). Furthermore, our results revealed that disease severity was very high in non-transgenic plants, and covering approximately 30% of the total leaf area than the *BjNPR1* transgenic lines at 15th dpi (**Figure 7B**). The disease resistance was assessed by measuring the average lesion diameter in the *Alternaria* infected leaves for both *BjNPR1* transgenic and non-transformed plants, and lesion diameter was 50% reduced in the former compared to non-transgenics (**Figure 7B**). We also observed that increased number of lesions spread on distal or non-infected leaves in non-transformed plants than *BjNPR1* transgenic plants after *Alternaria* infection (**Figure 7C**). Based on disease index (0–10 scale), highest disease incidence (3–4) was seen in control plants, while disease incidence 1–2 was seen in *BjNPR1* transgenic lines following infection (**Figure 7D**). Additionally, we also monitor the cell death and fungal biomass in transgenic and wild-type plants after *Alternaria* infection using trypan blue staining. Based on the microscopic examinations, the infection increased the number of dead cells with larger and expanding cell death areas observed beyond the inoculation site in non-transgenic plants compared to transgenic lines (**Figure 8A**). Moreover, the fungal biomass and spore load after 6th, 12th, and 15th of *Alternaria* infection were significantly low in transgenic lines than that of non-transgenic *B. juncea* plants (**Figure 8B**). Therefore, our results revealed that *BjNPR1* transgenic plants display partial resistance to *Alternaria* leaf blight as there was delay in lesion appearance, size, and spread of infection in comparison to non-transgenic plants.

### *BjNPR1* Plants Showed Improved Resistance against Powdery Mildew

**FIGURE 9:**
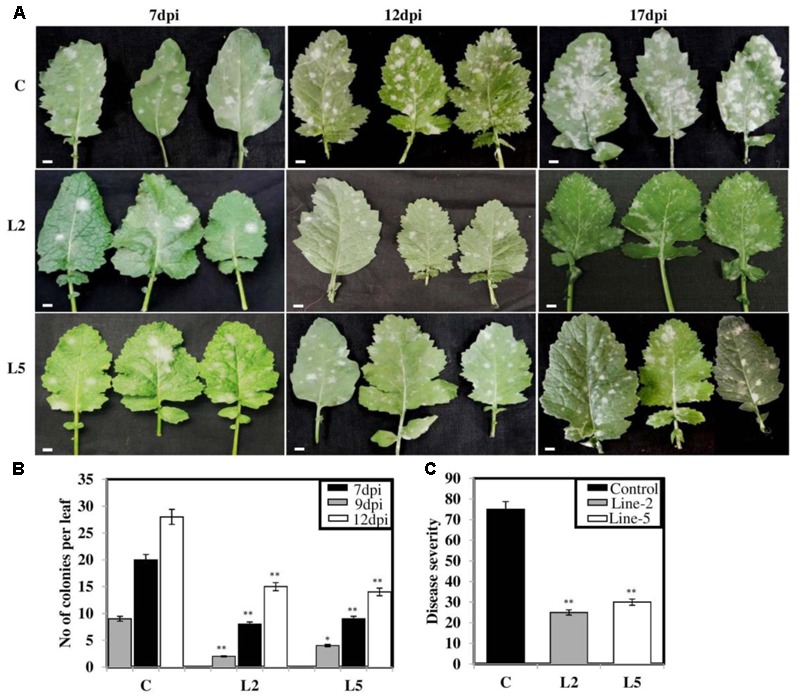
Screening of *BjNPR1* transgenic lines for powdery mildew disease resistance. Forty days old wild-type plants and *BjNPR1* transgenic plants were infected with *E. cruciferarum* and disease scoring was done at different time intervals. **(A,B)** *BjNPR1* transgenic lines (L2 and L5) showed reduced number of *E. cruciferarum* colonies than wild-type plants at 7th, 12th, and 18th dpi. **(C)** *E. cruciferarum* disease severity in *BjNPR1* transgenic lines and wild-type plants. Bar = 35 μm. The asterisks indicate statistically significant differences between the *BjNPR1* transgenic and control (non-transgenic) plants after powdery mildew infection (^∗^*P* < 0.05; ^∗∗^*P* < 0.01).

**FIGURE 10:**
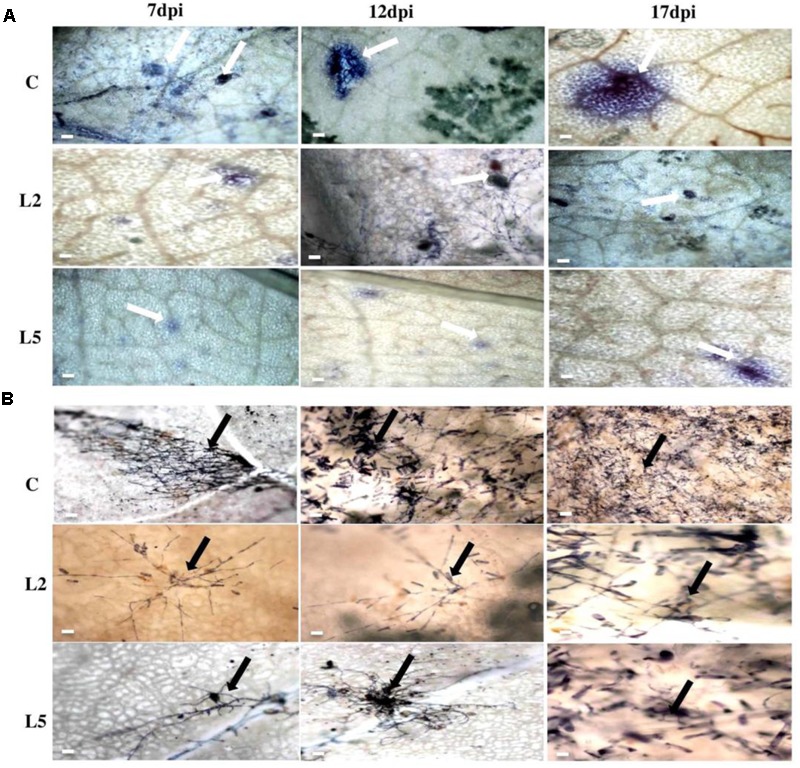
Microscopic examination of cell death and fungal biomass in *BjNPR1* transgenic and wild-type plants using trypan blue staining. **(A)** Microscopic examination of *E. cruciferarum* mediated cell death in *BjNPR1* lines and wild-type plants are shown with bold white arrows. **(B)** *E. cruciferarum* spore load or biomass in *BjNPR1* transgenic lines and wild-type plants at various dpi after trypan blue staining are highlighted with bold black arrows. Bar = 30 μm.

In the present study, we also examined the resistance level of the *BjNPR1* transgenic lines against powdery mildew disease, another important fungal disease of *B. juncea* caused by biotrophic pathogen (*E. cruciferarum*) which is entirely different in mode of infection style and signaling pathways from *A. brassicae*. To assess the resistance level of *BjNPR1* transgenic lines against powdery mildew, plants were infected and disease scoring was done for 1–3 weeks. In non-transgenic plants, higher number of *E. cruciferarum* colonies was observed than transgenic lines on 7th, 12th, and 17th day after infection (**Figure 9A**). Based on colony count, there was approximately 50% reduction in newly formed colonies between transgenic lines and non-transgenic plants (**Figure 9B**). At 17th day of infection, transgenic plants showed powdery mildew infection with a disease scale of 3–4 (30–40%), while as non-transformed leaves (wild type) revealed 7–8 (70–80%) of disease incidence, respectively (**Figure 9C**). In addition, *E. cruciferarum* mediated cell death was examined in *BjNPR1* transgenic and wild-type plants at different time points using trypan blue staining and light microscopy. Based on microscopic observations, more cell death was observed in control than that of transgenic plants (**Figure 10A**). To further investigate the role of *BjNPR1* in improving powdery mildew disease resistance, the growth or fungal biomass of *E. cruciferarum* in *BjNPR1* transgenic lines with wild-type plants was compared using light microscopy. As shown in **Figure 10B**, overexpression lines (L2 and L5) showed significant reduction of fungal biomass of *E. cruciferarum* than wild-type plants at 7th, 12th, and 17th dpi. However, overexpression of *BjNPR1* could not inhibit the growth of *E. cruciferarum* completely, thereby providing only partial resistance to powdery mildew disease. Also, more number of leaves and pods were infected in non-transgenic plants as compared to transgenic lines. These results indicate that *BjNPR1* transgenic plants exhibited partial resistance to powdery mildew infection, which was sufficient to delay the spread of infection in non-infected leaves or other parts.

## Discussion

Identification and understanding the role of defense regulatory genes is necessary to develop disease resistant transgenic crops in agricultural system. Manipulation of regulatory genes has many beneficial roles such as activation of multiple defense genes or pyramids which provides effective and long-lasting protection compared with a single gene approach. Hence, in the present study *BjNPR1* a regulatory gene was isolated and characterized, and phylogenetic analysis of the predicted *BjNPR1* protein with other known NPR1-like sequences revealed that they are grouped into distinct clades. However, *BjNPR1* fall within the same clade as other *Brassica* genus NPR1 proteins (**Figure 1**). Based on structural analysis, *BjNPR1* protein contains domains such as an ankyrin repeat domain and a BTB/POZ domain, which are highly conserved in all *NPR1* proteins (Cao et al., 1997; Kinkema et al., 2000; Mou et al., 2003). These domains are essential components of *NPR1* and provide functions relating to *NPR1*-dependant co-activation of TGA transcription factors and protein–protein binding (Cao et al., 1997; Rochon et al., 2006). A time course expression analysis of *BjNPR1* after defense hormonal treatments and fungal infections were carried out, and finally *BjNPR1* was overexpressed in *B. juncea* to exhibit disease resistance to *Alternaria* blight and powdery mildew.

Phytohormones, including SA, MeJA, ET, and ABA play an essential role in the regulation of plant immune responses to microbial pathogens. However, each signal molecule or pathogen has its specific mechanism (Kunkel and Brooks, 2002). It is well documented that SA signaling pathway is involved in the induction of SAR while as JA/ET are involved in the activation of induced systemic resistance. *NPR1* (a regulatory protein) is not only a bonafide receptor of SA but also a positive regulator of SAR, and plays a vital role in SA/JA signaling crosstalk (Spoel et al., 2003; Li et al., 2004; Wu et al., 2012). Mutant npr1-1 plants are not only compromised in SAR but also in basal resistance against many types of pathogens that are sensitive to SA dependent defenses (Dong, 2004). Previous studies have revealed that *NPR1* plays a central role in the induced defense signaling network that is controlled by SA, JA, and ET (Dong, 2004; Pieterse and Van Loon, 2004). There is plethora of studies on defense signaling cascades, but most of them have been carried out in model plants. Therefore, uncovering the role of SA/JA master regulator (*BjPNR1*) in *B. juncea* will provide novel insights at molecular level. Previously, the expression kinetics of *NPR1* or its homologs was found to be increased significantly after exogenous application of SA that leads to the activation of SAR (Mou et al., 2003; Yuan et al., 2007). Exogenous application of SA not only increases *NPR1* transcript accumulation but also changes its protein architecture in the nucleus, mainly through posttranslational modifications (Mou et al., 2003; Tada et al., 2008). *NPR1* and *TGA1* are crucial redox-controlled regulators of SAR in plants. Generally, *NPR1* is found as an oligomer within the cytoplasm of uninduced cells and changes in SA concentration lead to an altered redox environment within the cell, leading the nuclear localization of *NPR1* in its monomeric form (Mou et al., 2003). *NPR1* monomers interact with the reduced form of *TGA1*, which targets the activation sequence-1 (as-1) element of the promoter region of defense proteins (Després et al., 2003). In addition, SA-mediated redox modulation also plays an important role in the SA-mediated attenuation of the JA signaling pathway (Koornneef and Pieterse, 2008). In this study, we found that SA increased transcript levels of *BjNPR1* in *B. juncea* (**Figure 3A**), consistent with results observed in different crop plants (Zhang et al., 2008). On the other hand, exogenous treatment with JA did not alter the expression of *BjNPR1* (**Figure 3B**), similar results were also observed in avocado plants (Backer et al., 2015). However, contradictory results were seen in rice and banana seems to be host specific interactions (Yuan et al., 2007; Endah et al., 2008). Generally, ABA not only plays a central role in abiotic stress signal transduction, but also has been known to have positive or negative impact on plant immune system (Fan et al., 2005; de Torres–Zabala et al., 2007). Previous reports have shown that ABA promotes *NPR1* degradation in *Arabidopsis*. In this study, exogenous application of ABA decreases the expression of *BjNPR1* when compared to mock treated plants (**Figure 3C**). Many reports have shown that ABA appears upstream of *NPR1* and suppresses the expression of both *NPR1-*dependent and independent signaling signatures. Our results further provide the evidence that ABA negatively regulates *NPR1*, positive regulator of SAR pathway in *B. juncea.* Altogether, our results revealed that *NPR1* is distinctly regulated by defense stimulators, and also confirms that *BjNPR1* is likely to be dependent on SA signaling which was consistent with the sequence analysis data that *BjNPR1* contained a nuclear localization signal (NLS1) that was critical for SA-mediated expression of *PR* genes.

There is growing body of evidences that SA signaling triggers resistance to biotrophic pathogens, whereas a JA/ET pathway induces resistance to necrotrophic pathogens (Glazebrook, 2005). In the present study, *BjNPR1* was moderately induced by necrotrophic pathogen (*A. brassicae*) (**Figure 3D**) and the expression seems to be JA independent as it was not induced during JA treatment. Mazumder et al. (2013) also reported that *A. brassicicola*, a necrotrophic pathogen increases SA accumulation in *B. juncea* during early stages of disease development which might suppress the JA pathway for successful infection. In our previous study, we have also observed the induction of SA marker gene *PR1* after *A. brassicae* infection (data not shown). These results suggest that there is a hormonal crosstalk in *B. juncea* during *Alternaria* infection which could trigger the expression of *NPR1* or SA dependent genes. As expected, transcript levels of *BjNPR1* were significantly increased during *E. cruciferarum* infection as compared to uninfected plants (**Figure 3E**), similar to that observed by Dai et al. (2016). Furthermore, the expression levels of *BjNPR1* in powdery mildew infected plants were relatively higher than that of *Alternaria* infected plants because pathogens causing powdery mildew disease are known as strict biotrophic pathogens which rely on SA pathway (Oliver and Ipcho, 2004).

Previous studies have revealed that *Arabidopsis NPR1* when transformed into different crop plants showed enhanced disease resistance which make *NPR1* a promising and potential candidate gene for developing disease resistant transgenic plants. In this study, *B. juncea* transgenic plants were generated by overexpressing *BjNPR1* using *35S* promoter through *Agrobacterium* mediated plant transformation. It was earlier reported that overexpression of *AtNPR1* or its homolog *OsNH1* in rice transgenic lines although improved resistance to pathogens but showed many detrimental effects such as chlorotic lesions, hypersensitive to light and produced higher amount of reactive oxygen species which leads cell death (Fitzgerald et al., 2004; Chern et al., 2005). However, in our study *BjNPR1* transgenic plants exhibited normal phenotypes and did not showed any abnormalities, and similar findings were also observed in wheat, tobacco, and apple *NPR1* transgenic lines. We next addressed whether *BjNPR1* transgenic lines could activate SA or JA defense pathways by increasing the accumulation of defense marker genes (*PR* genes). As many studies have revealed that transgenic plants overexpressing *AtNPR1* activates *PR* gene expression in tomato, grape, tobacco, and rice (Lin et al., 2004; Chern et al., 2005; Zhang et al., 2010; Le Henanff et al., 2011). However, contradictory results were also seen in carrot plants where *NPR1* overexpression lines did not increase the transcript levels of *PR* genes under normal conditions. In present study, overexpression of *BjNPR1* significantly increases the transcript levels of SA dependent *PR* genes like *BjPR1, BjPR2*, and *BjPR5*. However, low induction of JA signature (PR) genes was observed in *BjNPR1* transgenic plants (**Figure 5**). NPR1 has been demonstrated to be an important transducer of the SA signal in the SA-mediated activation of *PR* gene expression and broad-spectrum resistance (Cao et al., 1994). Most of these PR proteins possess antifungal activity, and contribute effective and broad spectrum of disease resistance in *BjNPR1* transgenic lines. The expression of *BjPR* genes in *BjNPR1* transgenics further reveals that *NPR1* activates SAR in *B. juncea*, an immune response effective against multiple pathogens. Interestingly, microarray analysis in *Arabidopsis* revealed that among SA-induced defense genes, more than 90% were *NPR1*-dependent genes (Wang et al., 2006; Blanco et al., 2009). On the other hand, *Arabidopsis* npr1 mutants are not responsive to SA, are compromised in their ability to express *PR* genes like *PR1, PR2*, and *PR5* (Liu et al., 2005).

Many reports have revealed that *NPR1* confers resistance to both necrotrophs and biotrophs (Cao et al., 1997; Friedrich et al., 2001; Makandar et al., 2006; Malnoy et al., 2007; Zhang et al., 2010) which led to the proposal of introduce and overexpression of *BjNPR1* as a promising candidate gene for engineering broad-spectrum disease resistance in *B. juncea*. To evaluate the role of *BjNPR1* in disease resistance, two lines (L2 and L5) were chosen for disease screening against *Alternaria* and powdery mildew infection. Recently, overexpression of *NPR1* in peanut was reported to lead enhanced disease resistance against fungal pathogens (Sundaresha et al., 2016). Consistent with these reports, the results of our study revealed that overexpression of *BjNPR1* in *B. juncea* leads partial disease resistance against both necrotrophic (*A. brassicae*) and biotrophic (*E. cruciferarum*) fungal pathogen, as transgenic plants showed delayed symptoms, reduced mean lesion diameter, number of colonies and disease spreading to distal/non-infected parts of the plant (**Figures 7, 8**). These results provide the evidence that constitutive expression of *NPR1* in *B. juncea* showed high alertness in distal leaves (in the form of SAR) for subsequent infections at least in the early stages of infection. Our results also revealed that overexpression of *BjNPR1* delayed the onset of *Alternaria* and powdery mildew disease therefore displays partial not complete resistance in *B. juncea.* Consistent with our reports, overexpression of *AtNPR1* in carrot plants led enhanced disease resistance to biotrophic and necrotrophic fungal pathogens (Wally et al., 2009; Zhang et al., 2010). Many studies have shown that NPR1 or NPR1-like proteins confer resistance against fungal and bacterial pathogens, and this resistance is related to *PR* gene expression in transgenic plants. In present study, overexpression of *BjNPR1* significantly increases the transcript levels of SA dependent *PR* genes like *BjPR1, BjPR2*, and *BjPR5* which are universally known to posses potential antifungal activity. Previous studies have also shown that constitutive high-level expression of PR1, PR2, and PR5 in transgenic plants conferred tolerance to infection (Alexander et al., 1993; Liu et al., 2012; Gupta et al., 2013).

In summary, overexpression of *BjNPR1* into *B. juncea* imparts disease resistance to two economically important fungal pathogens, thereby supporting results from previous studies on *NPR1* transgenic carrot (Wally et al., 2009); cotton (Parkhi et al., 2010), and peanut (Sundaresha et al., 2016). Differences in the degree and development of disease symptoms caused by *A. brassicae* and *E. cruciferarum* between *BjNPR1* and untransformed plants were clearly observed. The disease resistance of *BjNPR1* transgenic *B. juncea* exposed to fungal pathogens may be due to the protection conferred by the accumulation of *PR* genes and SA mediated activation of SAR. However, in *Arabidopsis, AtNPR1* also is associated with the activation of systemic defenses that are independent of SA (Pieterse et al., 1998). Future studies will be carried out to ensure the overall efficiency of disease resistance of overexpression of *BjNPR1* transgenic plants under field conditions. Our future study would also focus on exploring the role of *BjNPR1* against combined biotic and abiotic stresses in *B. juncea* which is the theme of future research. The results described in this study point to the need to further dissect the signaling pathways or knockout mutant studies will provide new insights into the precise functions of the BjNPR1 gene in regulating responses to biotic as well as in abiotic stress in *B. juncea.* Indeed, constitutive expression of *AtNPR1* in many crops was associated with dwarfing and the spontaneous development of lesions which was not observed in *BjNPR1* transgenic plants which could have limit their commercial qualities. However, considering the detrimental phenotypes associated with At*NPR1* expression in rice, the exploitation of *NPR1* for improving disease resistance in economic important crops should take into consideration the physiology of the transgenic plants. In conclusion, *BjNPR1* may serve as a potential candidate gene for developing disease resistant transgenic crops by genetic engineering.

## Author Contributions

AG conceived and designed the research. SA has performed all the experiments and wrote the manuscript. ZM, PY, and PP contributed in data analysis. AT and JB has contributed in bioinformatic analysis. HM, RM, and SR contributed in tissue culture. AG contributed in manuscript proofreading. All authors read and approved the manuscript.

## Conflict of Interest Statement

The authors declare that the research was conducted in the absence of any commercial or financial relationships that could be construed as a potential conflict of interest.

## Footnotes

1 http://www.ebi.ac.uk/clustalw/

2 http://pfam.xfam.org/

3 http://www.sbg.bio.ic.ac.uk/phyre2/html/page.cgi?id=index

4 http://www.csbio.sjtu.edu.cn/bioinf/Cell-PLoc/

## Supplementary Material

FIGURE S1 Shoot regeneration from the calli of hypocotyls in *Brassica juncea*. **(A)** *B. juncea* plantlets grown in magenta boxes. **(B)** Hypocotyls on pre-culture media. **(C)** Explants on co-cultivation media. **(D)** Explants on selective media. **(E)** Explants grown on shoot induction media. **(F,G)** Explants grown in shoot regeneration media. **(H,I)** Explants grown in rooting media. **(J)** Transgenic to plant grown in soil-rite for hardening. Scale bars = 1 cm.
