# Supplementary figures and images for: Overexpression of NPR1 in Brassica juncea Confers Broad Spectrum Resistance to Fungal Pathogens
Source: Front Plant Sci. 2017 Oct 4;8:1693. doi: 10.3389/fpls.2017.01693 (PMC5632730; doi:10.3389/fpls.2017.01693)

**Figure S1**

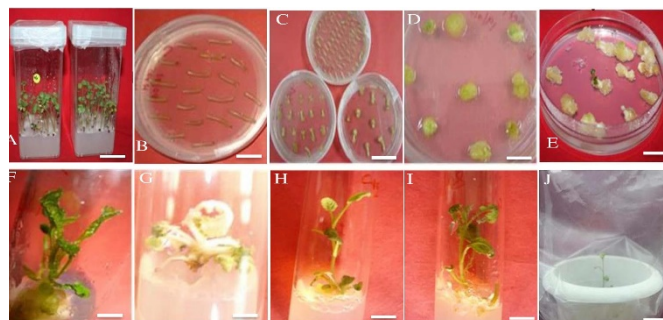

Supplement: FIGURE S1 — Shoot regeneration from the calli of hypocotyls in Brassica juncea. (A) B. juncea plantlets grown in magenta boxes. (B) Hypocotyls on pre-culture media. (C) Explants on co-cultivation media. (D) Explants on selective media. (E) Explants grown on shoot induction media. (F,G) Explants grown in shoot regeneration media. (H,I) Explants grown in rooting media. (J) Transgenic to plant grown in soil-rite for hardening. Scale bars = 1 cm. [file Image_1.pdf]
