# Supplementary material for: Overexpression of NPR1 in Brassica juncea Confers Broad Spectrum Resistance to Fungal Pathogens
Source: Front Plant Sci. 2017 Oct 4;8:1693. doi: 10.3389/fpls.2017.01693 (PMC5632730; doi:10.3389/fpls.2017.01693)
Supplement: Supplementary file 2 [file Table_1.pdf]

TABLE S1 Overall transformation efficiency in *B. juncea* using co-cultivation method

**No of Batches**	**No of hypocotyls transformed**	**Positive plants obtained**	**Transformation Efficiency (%)**
23	1080	15	2.71
